# Annotations

**Published:** 1890-05-10

**Authors:** 


### Annotations.

**What is an Expert?**

It is often said that medical witnesses are of no value, because if six of them aver that a thing is white, half-a-dozen can easily be found who will quite as solemnly swear that it is black. Judging from recent decisions purely scientific witnesses seem to be falling into the same general disrepute. A few days ago, a Merton dairyman was charged with selling milk from which 30 per cent. of its original fat had been abstracted. Professor Redwood spoke on behalf of the defendant, and argued that if a churn full of milk remained stationary for three quarters of an hour the cream would rapidly rise to the surface, and the milk at the bottom of the churn would thus become skimmed milk. It appears that tl e milk, which was the subject of the charge, had been standing in the churn for a considerable time, and that thus part of its cream had naturally and necessarily risen to the top. It was also stated by Professor Redwood, that milk which is moved from a warm place to a cooler one throws up its cream much more rapidly. Thus far expert number one. The inspector who had brought the charge called another expert in the person of Dr. Stevenson. Dr. Stevenson did not agree with the opinion expressed by Dr. Redwood. He admitted that the cream would rise when the milk was brought into a cold atmosphere from a warm one, but it would not rise anything like 30 per cent. Thus, expert number two. The magistrate said he had had the opinions of two experts; he himself would not express an opinion. But though he would not express his view of the matter in words, he showed unmistakeably what his opinion was, at least concerning the experts, for he promptly dismissed the summons. Now the writer happens to have had a good deal of experience of dairies and churns, of milk and of cream. His view of the particular point at issue would be this, that he would rather have the opinion of a clever dairy-maid than of all the scientific professors in London put together. No doubt if a qualitative and quantitative analysis had been required both the experts would have been quite at home; but in such a matter as the rapidity of the rising of the cream under varying circumstances, a capable dairy-maid would have beaten them both hollow. We do not complain of this fact at all; but we do complain that those eminent scientific professors had not the good sense and candour to admit their own want of practical experience and competence in the case at issue.

**Doctors and Drink.**

The other day a medical man, fifty-five years of age, was charged at one of the London Police Courts with having made a false statement for the purpose of obtaining relief at the City of London Union. The inquiries of the police resulted in proving however that the prisoner's statement was not false, but quite correct. The magistrate thereupon discharged him, and gave him the amount of his railway fare to Dublin from the poor box. In the course of the hearing of the case it was stated that the prisoner's downfall was due to drink. But he, himself, affirmed that first an attack of fever and then an apoplectic fit had destroyed his memory, and rendered him incapable of practice. Be the facts as they might, this melancholy case serves to call public attention to the exhausting labours of certain classes of medical men, and to the consequent depression of their physical and mental energies, whereby a craving for strong drink is induced. If people will consider one or two facts about family doctors, they will have some understanding of the exhaustion of brain and body from which they are so liable to suffer. Those facts are that family doctors work seven days a week all the year round, and during a considerable portion of the year they work in the night as well as in the day. No non-medical person can understand at all what this signifies to the doctor. Even the general practitioner himself is not fully conscious of the harm his work is doing him whilst he is still at it. The only person who can really take the true measure of the case is the man who has spent ten, fifteen, or twenty years in general practice, and has afterwards been enabled to give it up and to spend his days and nights, his weekdays and his Sundays, in the same way as the ordinary man of business. Such a man knows how profound is the depression that seizes upon the family doctor almost every day during a great part of the year; how terrible is the temptation to get rid of it by alcoholic stimulants; how the diminution of spirit and energy, and even of memory, steadily progresses, and makes the victim feel that he is in danger of becoming helpless and old almost before middle life is reached. The man who at this crisis escapes from general practice, and renews both youth and health by normal living, regards with the deepest sympathy even those degraded medical men who have fallen into drunkenness and despair. The competition of the times affects family doctors more than any other class of persons whatsoever. The reason is plain: they can never "shut up shop" either day or night, weekday or Sunday. The merchant leaves his office at a certain hour of the evening, and does not return to it until the following morning. The shopkeeper closes his shop, even though it be late, and does not open it again until at least nine or ten hours of quiet have been enjoyed. But the doctor, as we have said, is practically always at business. People think that they are entitled to consult him as freely at nine in the evening as at niue in the morning, and at midnight as at mid-day. The moral of our little sermon is this—that all who possibly can should see the family doctor in the day time, and that all who are able to afford it should treat him more liberally in the matter of fees.

**Prescriptions in the Mother Tongue.**

A small pamphlet on the new drug Exalgine has been forwarded to us, and we presume to the medical profession generally. The writer of the pamphlet gives very careful instructions about the methods of using the drug. He prints at length an ideal prescription; and even though the pamphlet is addressed to doctors, every word of the prescription is in the English language. From a medical point of view, this is an important sign of the times. It has been the custom for a longer period than we care to definitely ascertain, for physicians to write their prescriptions in the Latin tongue. They themselves have much valued the custom in past times. To have asked a physician of fifty years ago to write a prescription in English would have given the good man a very decided nervous shock, unless he had happened to be an experienced man of the world, which he seldom was. Even now many very estimable doctors would think the glory had departed from their consulting-rooms if the Latin tongue were banished in favour of the vernacular. Patients, too, it must be admitted, have been known who have felt that a wondrous virtue lay hidden in hieroglyphics which the vulgar could not understand. Hop tea, for example, is a very homely beverage, but *decoctum humuli lupuli* is clearly a fluid of much more imposing dignity. Nevertheless, as any schoolboy knows, the two are identical. Patients in these times, as well as doctors, are beginning to understand thac there is but little virtue in a word or a name. Even grammarians no longer write Latin Grammars for English boys in the Latin tongue. They would be considered demented if they did. The Latin prescription seems to be going the way of the Latin medical text-book and of the grammarian's Latin rules. It is a hopeful indication: a movement in the direction of simplicity, scientific truthfulness, and academic good breeding. There is only, perhaps, one word to be said in defence of retaining the Latin usage in prescriptions; and that is that English and foreign chemists can alike understand the classical tongue. As a matter of experience, however, that is not always found to be the case; and even if it were the advantages are not nearly so great as are often supposed. They are, in fact, insignificant in comparison with those which might be enumerated in favour of the universal employment of the mother tongue for prescriptions. The chief among these are, of course, two; first, the avoidance of mistakes in dispensing, arising from the illegible handwriting of the physician, or the smallness of the Latin acquirements of the dispensing chemist; and secondly, the knowledge which patients would obtain of the exact character of the medicine they were taking. This latter, we consider, would be invaluable to patients, inasmuch as it would enable the more intelligent of them gradually to acquire experience of the drugs which agreed with them and did them good, exactly as they now gain a knowledge of the suitability or otherwise of different kinds of food. Of course, many doctors will be alarmed at such a suggestion, as tending to diminish the need for their services. Such alarm is entirely without foundation. There is no doubt that as men come to apprehend more clearly the profound problems of physiology, and the potent nature of the substances used in physic, so will they learn to dread the dangers which might result from their own ignorance, and to seek with ever-increasing readiness and appreciation the skilled services of the medical expert.

